# Early Intervention, Big Savings: The Future of CKD Management in Thailand

**DOI:** 10.1016/j.ekir.2024.06.035

**Published:** 2024-06-27

**Authors:** Blake Angell, Vivekanand Jha

**Affiliations:** 1 The George Institute for Global Health, University of New South Wales, Sydney, Australia; 2 The George Institute for Global Health, University of New South Wales, New Delhi, India; 3 School of Public Health, Imperial College, London, UK; 4 Prasanna School of Public Health, Manipal Academy of Higher Education, Manipal, India

See Clinical Research on Page 2546

Chronic kidney disease (CKD) is largely preventable and treatable; however, its burden continues to grow, particularly in low- and middle-income nations.^1^ The global kidney health community has long advocated implementing CKD detection and treatment programs; however, robust health economic evidence to support such a strategy has been lacking. In this context, Paffett and colleagues provide estimates of the economic and health gains that could be realized across Thailand through the widespread implementation of an integrated care program to better manage CKD.^2^ Building on the ESCORT-1 and 2 trials conducted in rural Thailand, they use a cost-effectiveness analysis to project health system savings of almost US $6 billion within 5 years through the widespread implementation of an integrated care intervention to manage patients with mild-moderate CKD.^2^, ^3^, ^4^ Although these gains are modeled estimates, and several uncertainties remain, the work presents a strong case for investment to local policymakers. In doing so, it underscores the importance of locally developed health technology assessment (HTA) processes. Only by convincing policymakers of the need to invest in this program can these substantial health (and economic) gains be realized for the Thai population.

### CKD in Thailand and the Potential of Integrated Care

CKD is a significant and escalating public health issue in Thailand, with adult prevalence rates as high as 17.5% and a marked increase in the number of people requiring kidney replacement therapy, leading to a spiraling rise in health care expenditure.^5^^,^^6^ Out-of-pocket costs, a well-documented barrier to CKD care access globally and historically in Thailand, persist despite expanded care coverage under universal health coverage programs.^7^^,^^8^ Low CKD awareness levels have been a further barrier to care.^6^ The integrated care intervention, initially tested through the ESCORT-1 cluster randomized controlled trial, aimed to bring health care delivery closer to communities and intervene at earlier disease stages. Through earlier identification and intervention, the intervention significantly delayed the progression of CKD in a group of patients with stage 3 to 4 CKD living in a rural area about 400 km north of Bangkok.^4^ Using these data, and that from the follow-up ESCORT-2 prospective cohort study, which tracked similar patients receiving care through a more relaxed protocol to mirror the care they would receive in practice (outside a controlled trial context),^3^ Paffett *et al.*^2^ created a cost-utility model to estimate the health and economic impacts that widespread implementation of the intervention could have on patients and the Thai health care system. Their results are striking. They estimate that the integrated care intervention “dominates” existing care, with greater health benefits for the target population for lower care costs over the lifetime of the patient. Most remarkably, the authors estimate substantial savings to the Thai government’s health budget accruing very quickly after implementation, to almost US $6 billion over just 5 years, equivalent to about 7% of the Thai government's total health expenditure.

### Will These Benefits be Realized?

The academic literature has long recognized and extolled the virtues of early intervention and integrated care. However, the impacts of this in practice have been mixed. Many health care systems continue to invest heavily in later-stage, specialist-led care, whereas preventative, primary, and community-led interventions struggle to secure investments proportionate to their potential impact. Can we expect a different outcome in this case? There are many uncertainties inherent in any economic modeling study. Here, Paffett and colleagues have attempted to determine health and budgetary implications many decades down the track to assess them across the lifetimes of these patients. This is obviously a fraught exercise. The results are based on data from a relatively small, predominantly rural region; their applicability to other areas of the country is a major question. Can we expect the same magnitude of benefit and overall health impacts to be felt in the middle of Bangkok or even more remote areas of the country as were experienced in the 2 ESCORT studies? A key part of the intervention studied was the use of primary and community health care workers. Ensuring access to well-trained, available, and motivated community and primary health care workers to everyone across the country will be a major challenge, but one that is vital to achieving the results predicted here. Considering these uncertainties, the authors conduct a range of sensitivity analyses testing the impact of varying key model impacts on their findings. In this regard, their results are again promising, finding that the intervention will likely remain cost-effective even under significantly less optimistic assumptions. Ultimately, the largest factor in determining whether these benefits come to pass will be whether these studies are followed by the investment needed to implement the program across the health system.

### The Role of HTA

One reason optimism may be warranted is the HTA process developed to inform investments in the Thai health care system. HTA processes are systematic, multidisciplinary evaluations of the wide-ranging impacts of health technologies and interventions incorporating factors such as relative health outcomes, cost-effectiveness, budget impact, equity concerns, and more. Ideally, these frameworks serve as a conduit between research and policymaking, guiding investment decisions across health systems. Globally, there are many such systems, with probably the most well-known examples operating in high-income countries; examples are the National Institute for Health and Care Excellence in the United Kingdom, and Australia’s Medical Services Advisory Committee and Pharmaceutical Benefits Advisory Committee processes.^9^^,^S1,S2 Indeed, for many years after the inception of HTA processes in some high-income nations, HTA was perceived as a nice-to-have feature but one of secondary importance for less well-resourced health systems.S3

Fortunately, this perspective has evolved, and HTA is now recognized as a critical component of the prioritization process in countries across all income levels. Although all health systems struggle with limited budgets and the need to prioritize investment choices, these challenges are often more acute in less-resourced settings; and thus, the potential payoff from a well-functioning HTA system is larger in these contexts. This evolving understanding largely stems from recognizing the importance of local data, health systems, and preferences in informing local decisions. What is considered a good investment in 1 country may not be in another, even in situations where health care systems might appear quite similar in terms of resourcing or seemingly comparable populations. In this space, Thailand has emerged as a well-documented leader in the field, particularly among middle-income nations, with much of the work over recent years led by the Health Intervention and Technology Assessment Program.S4 Early studies have started demonstrating the impact that this has had on investment decisions in Thailand.S5-S7 Time will tell whether this intervention will join the others that have gained support from the HTA process and receives the investment needed to implement the program at scale.

### Conclusion

The work of Paffett *et al.*^2^ offers a powerful case for investment. Leaving aside the substantial health gains, very few health systems would turn down a potential US $6 billion worth of health care resources being freed up over just a few years. Although numerous uncertainties underlie these estimates, the biggest obstacle to achieving these large health gains is the initial investment required to scale the intervention across the country. In this regard, Thailand’s progress to becoming a global HTA leader offers promise that the evidence generated could inform investment decisions that could make a real difference to population health.

## Disclosure

BA is supported by a NHMRC Investigator Grant (GNT2010055). VJ has received grant funding from UK MRC, NIHR, and honoraria and consultancy fees from Bayer, Astra Zeneca, Visterra, Chinook, Vera, Biocryst, Otsuka, Baxter, Boehringer Ingelheim and ProKidney, under the policy of all payments being made to the organization.

## Supplementary Material

Supplementary file (PDF) Supplementary references.

## Footnotes

Supplementary File (PDF)
Supplementary References
