# Supplementary material for: Early Intervention, Big Savings: The Future of CKD Management in Thailand
Source: Kidney Int Rep. 2024 Jun 27;9(8):2323–5. doi: 10.1016/j.ekir.2024.06.035 (PMC11328743; doi:10.1016/j.ekir.2024.06.035)
Supplement: Supplementary file (PDF) — Supplementary references. [file mmc1.pdf]

Supplementary References

S1. Department of Health and Aged Care. Medical Services Advisory Committee. Accessed June 14, 2024. http://www.msac.gov.au/

S2. Department of Health and Aged Care. Pharmaceutical Benefits Scheme (PBS). Accessed June 14, 2024. https://www.pbs.gov.au/pbs/home

S3. Teerawattananon Y, Painter C, Dabak S, et al. Avoiding health technology assessment: a global survey of reasons for not using health technology assessment in decision making. Cost Effectiveness and Resource Allocation. 2021;19:1-8.

S4. HITAP. Health Technology and Assessment Program. Published 2014. Accessed June 16, 2024. https://www.hitap.net/en/

S5. Teerawattananon Y, Vishwanath Dabak S, Culyer A, Mills A, Kingkaew P, Isaranuwatchai W. Fifteen Lessons from Fifteen Years of the Health Intervention and Technology Assessment Program in Thailand. Health Systems & Reform. 2023;9(3):2330974.

S6. Tanvejsilp P, Taychakhoonavudh S, Chaikledkaew U, Chaiyakunapruk N, Ngorsuraches S. Revisiting Roles of Health Technology Assessment on Drug Policy in Universal Health Coverage in Thailand: Where Are We? And What Is Next? Value in Health Regional Issues. 2019;18:78-82. doi:10.1016/j.vhri.2018.11.004

S7. Mohara A, Youngkong S, Velasco RP, et al. Using health technology assessment for informing coverage decisions in Thailand. Journal of comparative effectiveness research. 2012;1(2):137-146.
